# Adverse Childhood Experiences, Health‐Related Quality of Life, and Chronic Disease Risks in Rural Areas of the United States

**DOI:** 10.1155/2018/7151297

**Published:** 2018-07-11

**Authors:** Amy Chanlongbutra, Gopal K. Singh, Curt D. Mueller

**Affiliations:** ^1^Federal Office of Rural Health Policy, Health Resources and Services Administration, US Department of Health and Human Services, 5600 Fishers Lane, Rockville, MD 20857, USA; ^2^Office of Health Equity, Health Resources and Services Administration, US Department of Health and Human Services, 5600 Fishers Lane, Rockville, MD 20857, USA

## Abstract

Exposure to adverse childhood experiences (ACEs) is associated with increased odds of high‐risk behaviors and adverse health outcomes. This study examined whether ACE exposure among individuals living in rural areas of the United States is associated with adult activity limitations, self‐reported general poor health status, chronic diseases, and poor mental health. Data from the 2011 and 2012 Behavioral Risk Factor Surveillance System (BRFSS) (N=79,810) from nine states were used to calculate the prevalence of ACEs in rural and urban areas. ACE scores were determined by summing 11 survey items. Multiple logistic regression was used to examine the association between ACE scores and health outcomes, including self‐reported general health status, chronic diseases, and health‐related quality of life. Approximately 55.4% of rural respondents aged ≥18 years reported at least one ACE and 14.7% reported experiencing ≥4 ACEs in their childhood, compared to 59.5% of urban residents who reported at least one ACE and 15.5% reporting ≥4 ACEs. After adjusting for sociodemographic covariates, compared to rural respondents who never reported an ACE, rural respondents who experienced ≥1 ACEs had increased odds of reporting fair/poor general health, activity limitations, and heart disease, which is consistent with previous studies. The odds of experiencing a heart attack were higher for rural residents reporting 2 and ≥4 ACEs; the odds of diabetes were higher for those with 3 ACEs; and the odds of ever having asthma or poor mental health was higher for those with ≥3 ACEs. Although individuals in rural areas are less likely to experience ACEs, over half of rural respondents reported experiencing an ACE in childhood. Programs aimed at preventing ACEs, including child maltreatment, can benefit rural areas by reducing adult morbidity and increasing quality of life.

## 1. Introduction

**Figure 1:**
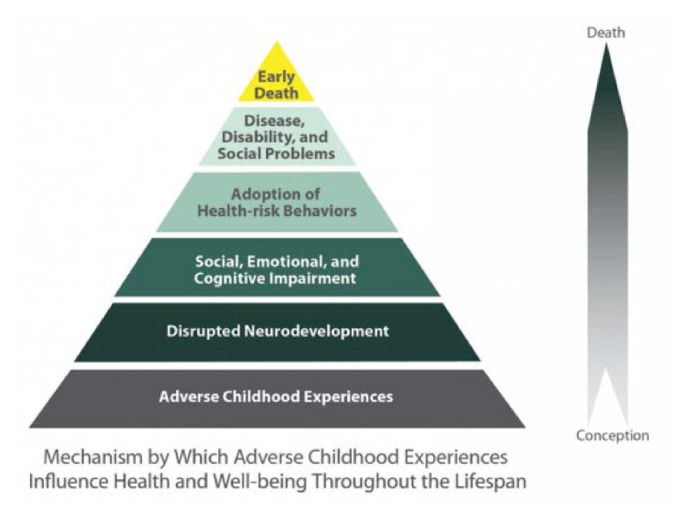
Conceptual model of the effects of ACEs on health and well-being.** Source**: Figure 1 is reproduced from [5].

Adverse childhood experiences (ACEs) are childhood abuses and household disruptions experienced before the age of 18 that includes exposure to mental illness, substance abuse, imprisonment, separation or divorce, adult violence, physical abuse, and sexual abuse [1–3]. Studies have shown that, compared to individuals who have never reported an ACE, individuals who experienced ACEs are at increased odds of high‐risk behaviors such as binge drinking, risky sexual behavior, and smoking as well as adverse health outcomes such as premature death, diabetes, stroke, depression, fair/poor health, myocardial infarction, asthma, disability, severe obesity, mental distress, and ever having a sexually transmitted disease [1, 3–5]. Moreover, exposure to one ACE can increase the odds of exposure to additional ACEs, indicating a relationship among other ACE exposures [6]. According to a recent study, over half of rural residents reported one or more ACE exposures, but after adjusting for sociodemographic factors, the odds of experiencing high ACE scores were not significantly different between urban and rural groups [2]. An early influential CDC‐Kaiser Permanente adverse childhood experiences (ACE) study developed a conceptual model outlining the impact of ACEs on development of an individual from conception to death, influencing neurodevelopment, development of health‐risk behaviors, and poor health outcomes later in life (Figure 1) [5]. The objective of this study is to determine whether ACE exposure among individuals living in rural areas is associated with increased odds of adult activity limitations, self‐reported general poor health status, chronic diseases, and poor mental health. This study contributes to the existing literature on health effects of ACEs by conducting research among a large sample of rural and urban residents from nine US states.

## 2. Methods

### 2.1. Study Sample

Analysis was completed using data from the 2011 and 2012 Behavioral Risk Factor Surveillance System (BRFSS), a state surveillance system that collects health practices and behavioral risk factors of noninstitutionalized US adults 18 and older through telephone interviews [7, 8]. The BRFSS has been conducted by the Centers for Disease Control and Prevention since 1984. The BRFSS conducts more than 400,000 adult interviews in the US about people's health related behaviors, chronic health conditions, and use of preventive health services, making it the largest continuously conducted health survey system in the world [9].

The present study included 982,154 participants who completed the 2011 and 2012 BRFSS interview and, among those who were interviewed, 79,810 individuals aged ≥18 years from nine states (Iowa, Minnesota, Montana, North Carolina, Oklahoma, Tennessee, Vermont, Washington, and Wisconsin) which administered the optional ACE module. Of those who answered the ACE module, 29,521 individuals lived in rural areas and 36,141 individuals lived in urban areas. In the BRFSS, respondents who lived in nonmetropolitan counties were classified as rural residents, whereas those living in suburbs, central cities, and outside central cities of metropolitan counties were classified as urban residents [7, 8].

### 2.2. Measures

Exposure to ACES was the primary independent variable in our study and is defined by the BRFSS ACE module through 11 questions:

1. Do you live with anyone who was depressed, mentally ill, or suicidal?
2. Did you live with anyone who was a problem drinker or alcoholic?
3. Did you live with anyone who used illegal street drugs or who abused prescription medications?
4. Did you live with anyone who served time or was sentence to serve time in a prison, jail, or other correctional facility?
5. Were your parents separated or divorced?
6. How often did your parents or adults in your home ever slap, hit, kick, punch or beat each other up?
7. Before age 18, how often did a parent or adult in your home ever hit, beat, kick, or physically hurt you in any way?
8. How often did a parent or adult in your home ever swear at you, insult you, or put you down?
9. How often did anyone at least 5 years older than you or an adult, ever touch you sexually?
10. How often did anyone at least 5 years older than you or an adult, try to make you touch them sexually?
11. How often did anyone at least 5 years older than you or an adult, force you to have sex?

Respondent answers for the each of the ACE questions were summed and then categorized into five ACE‐score categories: 0, 1, 2, 3, and ≥4. The dependent variables included activity limitation, general health status, number of poor mental health days (a health‐related quality of life outcome), and whether or not respondents were ever told by a health professional that they had any of the following conditions: diabetes, stroke, heart attack, angina or coronary heart disease, and asthma. The respondents were classified as having poor mental health if they had 14 or more days of poor mental health during the past 30 days, including stress, depression, and problems with emotions. The covariates included age, sex, race/ethnicity, veteran status, marital status, education, and family income. All variables were measured as shown in Tables 1 and 2.

### 2.3. Statistical Analysis

Prevalence estimates for sociodemographic characteristics were computed by geography and by ACE score‐categories using survey sampling weights that took into consideration the complex survey design of the BRFSS. Chi‐square statistics were used to examine differences in ACE scores by sociodemographic characteristics. Logistic regression was used to examine the relationship between health outcomes and ACE scores in rural and urban areas separately, controlling for relevant socio‐demographic variables. Sociodemographic variables that were included in the model had a p value less than 0.25 in the bivariate analysis. A p value of <0.05 was considered to be statistically significant.

Analyses were conducted by using SAS 9.3 [10] and SUDAAN 11.0.1 [11].

## 3. Results

Table 1 presents sociodemographic composition of the rural and urban populations. The rural population tended to be older than the urban population, with 27.0% of the rural respondents being aged 65 years and older compared with 23.5% of the urban respondents. Rural areas had a lower percentage of ethnic‐minority population than urban areas (11.6% versus 18.5%). Rural respondents had lower levels of education and income than their urban counterparts. Approximately 15.2% of rural respondents had less than a high school education, compared with 10.1% of urban respondents. About 18.8% of rural respondents were college graduates, compared with 30.1% of urban respondents.

Approximately 30.8% of rural respondents had less than $25,000 annual family incomes, compared with 22.1% of urban respondents.

**Figure 2:**
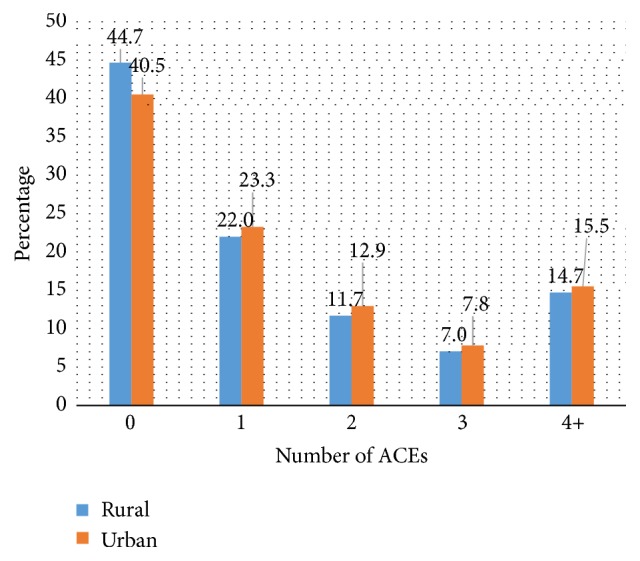
Prevalence of adverse childhood experiences (ACEs) in rural and urban areas of the United States, 2011-2012.** Source**: derived from the 2011-2012 Behavioral Risk Factor Surveillance System (BRFSS), 9 States.

Rural areas had significantly higher percentage of individuals without ACEs (i.e., with 0 ACE scores) (44.7%) compared with individuals living in urban areas (40.5%) [Figure 2]. Similarly, rural areas had lower percentage of individuals with ACE scores ≥4 (14.7%) compared with urban areas (15.5%) (Figure 2). The most prevalent ACE component for both rural and urban respondents was “being sworn at, insulted, or put down by parents or adults in the home” (31% versus 33% once/more than once), while living with alcoholic or problem drinkers and experiencing parental separation/divorce were the second and third most common responses for rural and urban areas (Table 1).

A higher ACE score in rural areas was significantly associated with older age, being female, being an ethnic minority, being divorced/never married/unmarried, having less education, lower income, and being out of work/unable to work (Table 2). Increasing ACE score was indicative of increasing prevalence and odds of activity limitations, fair/poor health, ever having a heart attack diagnosis, ever having angina or coronary heart disease, ever having had asthma, and poor mental health for urban and rural areas (Table 3). More specifically, exposure to one, two, three, and ≥4 ACEs in rural areas was significantly associated with greater odds of activity limitation due to physical, mental, or emotional problems, general health rated as fair/poor, and ever having had angina or coronary heart disease.

Compared to those who did not experience any ACEs, those experiencing 3 and ≥4 ACEs had, respectively, 1.9 and 2.7 times higher adjusted odds of reporting activity limitation. Those who experienced ≥4 ACEs had 2.0 times higher adjusted odds of rating their general health status as fair/poor than those who did not experience any ACEs. Compared to those who did not experience any ACEs, those experiencing 3 and ≥4 ACEs had, respectively, 1.5 and 2.4 times higher adjusted odds of coronary heart disease.

Those reporting 3 ACEs in rural areas had 36% higher adjusted odds of having diabetes than those who did not report experiencing any ACEs. Rural respondents experiencing 3 and ≥4 ACEs had, respectively, 62% and 90% higher adjusted odds of poor mental health compared to those reporting no ACEs. Rural respondents who reported 2 and ≥4 ACEs had, respectively, 35% and 94% higher adjusted odds of ever diagnosed with a heart attack than those reporting no ACEs. A somewhat similar but more pronounced association between higher ACE score and health outcomes was seen in urban areas compared to rural areas. In urban areas, activity limitation, fair/poor general health status, ever being diagnosed with diabetes, ever having a stroke, ever having a heart attack, ever having angina or coronary heart disease, ever having asthma, and poor mental health were all significantly associated with ≥4 ACEs (Table 3). One difference between rural and urban associations was that urban areas had a more consistent dose‐response effect of ACEs on ever having diabetes and ever having asthma than rural areas.

## 4. Discussion

The results indicate that a greater dose of ACE exposure corresponds to increased odds of an adverse health outcome after controlling for a number of sociodemographic variables. A dose‐response relationship is present as an increasing ACE score corresponds to higher likelihood of fair/poor general health, poor mental health, activity limitation, and chronic disease morbidity for both rural and urban areas. Results of this study are consistent with previous studies that indicate significant associations between exposure to ACEs and physical health outcomes (e.g., respiratory disease, cardiac disease, cancer, and mental health) [12–15]. The distribution of ACE score from 0 ACEs to ≥4 ACEs in rural areas is similar to the study conducted by Maine Rural Health Research Center (MRHRC) [2]. In this study, 55.4% of rural adults had at least 1 ACE exposure, while the study conducted by MRHRC found 56.6% of rural adults with at least 1 ACE exposure. Dose-dependent relationship that was observed in our study corresponds with previous literature that also found an association between amount of exposure between adverse experiences and risky behaviors and increased likelihood of disease conditions [1, 3, 5]. An adverse experience such as abuse, whether is physical or sexual, and neglect can have an impact on adult emotional and behavioral health, increasing odds of suicidal thoughts as well as suicide attempts, anxiety, depression, issues with creating and maintaining healthy intimate relationships, illicit drug use, as well as delinquency and adult criminality [16–20]. This is a seminal national rural study that examines a broad range of health outcomes that include general health status, mental health problems, chronic diseases, and activity limitation, many of which were not analyzed in relation to ACEs in previous smaller‐scale studies.

### 4.1. Limitations

One of the limitations of this study is that the ACES module was optional for states, so not all states participated. Since the sample is drawn from states that do not contain large urban areas, comparisons between urban and rural areas may not be valid between rural areas and states with large cities (e.g., California, New York, and Texas). Secondly, this study is a cross‐sectional study; therefore, causality cannot be established between the independent and dependent variables. However, the fact that ACEs occurred in childhood indicates a temporal relationship between ACE exposure and health outcomes measured as of the survey date. Thirdly, the BRFSS includes self‐reported questions, which can introduce possible recall bias, particularly regarding events that occurred in childhood. A bias between young and older respondents may occur as younger respondents may have an easier time recalling events that occurred in childhood. Fourthly, although the ACE measure itself captures number of adverse events, it does not measure other nuances such as amount of exposure to specific adverse events during childhood. Lastly, although the ACE measure used in our study is fairly comprehensive and consists of 11 different survey items, the variables making up the ACE measure are equally weighted. A factor‐based ACE index that differentially weights various ACE components might be a better methodological approach, which should be explored in future research.

### 4.2. Conclusions

In this study, using a large, nationally representative sample survey, the BRFSS, we found marked effects of adverse childhood experiences on a number of health outcomes (e.g., self‐assessed general health, mental health, activity limitation, heart disease, stroke, diabetes, and asthma) among adults aged ≥18 years living in both rural and urban areas of the United States. Many of these health effects of ACEs were independent of those associated with contemporary socioeconomic and demographic characteristics. Although individuals in rural areas are less likely to experience ACEs, over half of rural respondents reported experiencing an ACE in childhood. Programs aimed at preventing ACEs, including child maltreatment, can benefit rural areas by reducing adult morbidity and increasing quality of life.

Prevention of ACE begins early in childhood and includes creating a safe and positive environment for rural children and families and a system that supports healthy families. Strengthening an early childhood system that is trauma‐informed includes home visiting, child and adult mental health services, child welfare system, and others [21]. Rural providers can also play an important role, screening for ACEs and connecting families to social services through well‐child visits [2]. In furthering rural research on ACEs, cohort studies that include rural populations can also better understand the association and development of adverse health outcomes due to ACEs. Additionally, interactions of ACEs with age and gender should be explored further to determine if there are differences in type and severity of health outcomes between younger and older and between male and female respondents.

## Figures and Tables

**Table 1:** Description of the study sample (unweighted n, weighted %), 2011-2012 BRFSS, 9 US states.

Demographic Characteristics	**Total**			**Rural**			**Urban**			
	Number	%	SE	Number	%	SE	Number	%	SE	p-value
**Age (years)**										

18-24	3,591	12.4	0.26	679	7.4	0.42	801	7.7	0.37	<.001

25-34	7,437	15.9	0.24	1,764	9.8	0.34	2,212	10.3	0.30	

35-44	9,945	16.9	0.24	2,965	14.9	0.40	4,498	16.9	0.35	

45-54	14,756	19.4	0.23	5,142	20.1	0.41	6,859	21.6	0.37	

55-64	18,511	16.7	0.19	7,549	20.8	0.37	8,946	20.0	0.32	

≥65	24,980	18.7	0.18	11,230	27.0	0.39	12,513	23.5	0.32	

**Sex**										

Male	32,387	48.3	0.31	11,656	46.4	0.52	13,659	45.1	0.45	0.0618

Female	47,423	51.7	0.31	17,865	53.6	0.52	22,482	54.9	0.45	

**Race/Ethnicity**										

White, non-Hispanic (NH)	67,962	80.1	0.26	26,081	88.4	0.34	30,693	81.5	0.38	<.001

Black, NH	4,257	8.8	0.18	655	4.2	0.22	2,505	8.4	0.25	

Asian, NH	797	2.3	0.11	71	0.5	0.09	514	3.1	0.18	

Native Hawaiian/Other Pacific Islander, N	120	0.1	0.03	44	0.1	0.03	52	0.2	0.06	

American Indian/Alaska Native, NH	1,695	1.5	0.07	1,113	2.1	0.12	321	1.1	0.11	

Other, NH	533	0.6	0.05	178	0.4	0.05	254	0.6	0.06	

Multirace, NH	1,303	1.4	0.07	459	1.1	0.10	550	1.2	0.10	

Hispanic	2,526	5.3	0.16	680	3.1	0.21	977	3.9	0.22	

**Veteran Status**										

Yes	10,455	11.6	0.18	4,076	13.0	0.32	4,937	12.8	0.27	0.603

No	69,323	88.4	0.18	25,435	87.0	0.32	31,189	87.2	0.27	

**Marital Status**										

Married	43,806	54.3	0.30	16,954	61.9	0.51	20,064	61.6	0.44	<.001

Divorced/Separated	12,731	12.7	0.18	4,501	11.8	0.30	5,828	11.0	0.23	

Widowed	10,191	6.8	0.11	4,701	9.7	0.22	5,020	8.3	0.18	

Never Married	10,712	22.3	0.30	2,701	14.1	0.47	4,288	16.2	0.42	

Unmarried Couple	2,096	3.9	0.13	587	2.5	0.18	789	2.9	0.17	

**Education**										

Never Attended School	93	0.2	0.03	40	0.3	0.06	32	0.2	0.04	<.001

<High School	5,898	12.2	0.24	2,559	14.9	0.46	2,243	9.9	0.33	

High School Graduate	23,177	30.1	0.28	9,954	35.2	0.49	9,305	27.7	0.41	

Some College	22,891	32.6	0.29	8,203	30.8	0.47	10,257	32.2	0.43	

College	27,614	24.8	0.23	8,714	18.8	0.33	14,241	30.1	0.37	

**Income**										

<$15,000	7,183	10.1	0.20	2,959	9.9	0.31	2,684	7.1	0.23	<.001

$15,000 to less than $25,000	12,666	19.0	0.27	5,213	20.9	0.47	5,028	15.0	0.36	

$25,000 to less than $35,000	9,052	12.9	0.22	3,695	14.5	0.38	3,833	12.0	0.31	

$35,000 to less than $50,000	11,272	15.7	0.23	4,525	17.5	0.40	4,825	15.0	0.33	

≥$50,000	29,225	42.4	0.31	9,290	37.1	0.52	14,793	50.9	0.48	

**Employment Status**										

Employed or Self-Employed	40,948	57.7	0.30	13,900	53.0	0.51	17,481	54.2	0.44	<.001

Out of Work	4,292	7.1	0.17	1,306	5.8	0.27	1,909	6.3	0.22	

Homemaker/Student	6,317	11.0	0.22	2,138	8.7	0.33	2,727	10.7	0.33	

Retired	22,790	17.9	0.18	9,902	24.8	0.38	11,624	22.8	0.32	

Unable to Work	5,264	6.3	0.14	2,192	7.8	0.27	2,314	5.9	0.20	

**Adverse Childhood Experiences (ACEs)**										

Live with Anyone Depressed, Mentally Ill, Suicidal										

Yes	12,447	17.0	0.24	3,978	13.7	0.37	5,597	15.9	0.34	<.001

No	66,727	83.0	0.24	25,304	86.3	0.37	30,254	84.2	0.34	

Live with problem drinker or alcoholic										

Yes	19,086	24.7	0.27	6,809	22.9	0.44	8,493	23.6	0.38	0.197

No	60,281	75.3	0.27	22,556	77.1	0.44	27,437	76.4	0.38	

Live with someone who used illegal street drugs or who abused prescription medications										

Yes	6,188	10.6	0.20	1,812	8.0	0.32	2,538	8.4	0.26	0.269

No	73,196	89.4	0.20	27,570	92.0	0.32	33,416	91.6	0.26	

Lived with Anyone who Served Time in Correctional Facility										

Yes	4,096	7.9	0.19	1,195	5.7	0.28	1,610	5.9	0.24	0.713

No	75,448	92.1	0.19	28,227	94.3	0.28	34,424	94.1	0.24	

Experienced Parental Separation or Divorce										

Yes	16,540	26.3	0.28	5,311	20.6	0.44	7,020	22.5	0.40	<.001

No	62,219	72.4	0.29	23,925	78.8	0.45	28,618	76.3	0.40	

Parents Not Married	591	1.2	0.08	127	0.6	0.07	291	1.2	0.12	

Witnessed Parents or Adults in Home Slap, Hit, Kick, Punch, or Beat Each Other										

Never	66,379	83.0	0.24	24,749	84.5	0.38	30,309	84.6	0.33	0.833

Once	3,092	4.5	0.13	1,069	4.2	0.21	1,403	4.3	0.19	

More than Once	9,015	12.6	0.21	3,203	11.3	0.33	3,839	11.1	0.28	

Being Slapped, Hit, Kicked, Punched or Beat by Parents or Adults in the Home										

Never	67,042	83.6	0.23	25,095	85.4	0.38	30,588	85.1	0.33	0.428

Once	2,976	4.0	0.12	1,002	3.6	0.20	1,337	4.0	0.18	

More than Once	8,997	12.4	0.21	3,111	11.0	0.34	3,892	10.9	0.29	

Being Sworn at, Insulted, or Put Down by Parents or Adults in the Home										

Never	53,335	65.2	0.30	20,444	69.5	0.49	24,326	66.9	0.43	0.000

Once	4,726	7.2	0.17	1,610	6.5	0.29	2,095	7.0	0.26	

More than Once	20,397	27.6	0.28	6,978	24.1	0.45	9,083	26.2	0.40	

Being Touched Sexually by Adult or Anyone at Least 5 Years Older										

Never	70,473	90.2	0.18	26,197	90.9	0.29	31,925	90.6	0.25	0.631

Once	2,959	3.5	0.11	1,042	3.4	0.19	1,400	3.5	0.15	

More than Once	5,238	6.3	0.15	1,861	5.7	0.23	2,311	6.0	0.20	

Being Made to Touch Sexually an Adult or Anyone at Least 5 Years Older than Respondents										

Never	72,950	92.7	0.16	27,119	93.6	0.26	33,118	93.4	0.21	0.580

Once	2,125	2.7	0.10	727	2.3	0.17	974	2.6	0.14	

More than Once	3,628	4.6	0.13	1,272	4.1	0.20	1,567	4.1	0.17	

Being Forced to Have Sex with An Adult or Anyone at Least 5 Years Older than Respondent										

Never	75,447	95.7	0.13	27,998	96.4	0.19	34,261	96.3	0.17	0.806

Once	1,115	1.6	0.08	388	1.2	0.11	466	1.3	0.10	

More than Once	2,142	2.8	0.10	731	2.3	0.16	927	2.4	0.13	

**Table 2:** Distribution of ACE scores by sociodemographic characteristics in rural and urban areas of the United States, 2011-2012 BRFSS, 9 states (unweighted n, weighted %).

**RURAL (n=29,521)**	**ACE Score 0** **n=13,521** **(%)**	**SE**	**ACE Score 1** **n=6,320** **(%)**	**SE**	**ACE Score 2** **n=3,446** **(%)**	**SE**	**ACE Score 3** **n=2,160** **(%)**	**SE**	**ACE Score 4+** **n=4,074** **(%)**	**SE**	**p-value**

**Age (years)**											

18-24	7.0	0.64	5.7	0.71	9.6	1.56	7.1	1.45	9.4	1.07	<.001

25-34	7.8	0.45	10.5	0.77	9.6	0.94	11.6	1.26	14.3	1.11	

35-44	10.8	0.50	16.2	0.92	16.6	1.22	20.1	1.65	21.7	1.25	

45-54	17.1	0.56	21.3	0.90	23.1	1.30	19.4	1.44	25.6	1.17	

55-64	21.3	0.57	19.8	0.76	21.4	1.13	23.7	1.41	19.2	0.99	

≥65	36.1	0.66	26.6	0.84	19.7	0.99	18.3	1.19	9.8	0.61	

**Sex**											

Male	47.3	0.76	49.2	1.09	47.2	1.60	45.5	1.89	39.3	1.44	<.001

Female	52.8	0.76	50.8	1.09	52.8	1.60	54.5	1.89	60.7	1.44	

**Race/Ethnicity**											

White, non-Hispanic (NH)	89.8	0.48	88.3	0.8	88.6	0.94	86.1	1.44	85.4	1.00	<.001

Black, NH	4.2	0.29	5.3	0.64	3.6	0.54	4.3	0.82	3.4	0.57	

Asian, NH	0.7	0.17	0.4	0.18	0.4	0.28	0.6	0.30	0.1	0.03	

Native Hawaiian/Other Pacific Islander, NH	0.1	0.02	0.1	0.03	0.1	0.07	0.1	0.10	0.3	0.18	

American Indian/Alaska Native, NH	1.5	0.12	1.7	0.21	2.3	0.36	4.5	0.91	3.7	0.38	

Other, NH	0.3	0.06	0.5	0.11	0.3	0.12	0.3	0.09	0.7	0.18	

Multirace, NH	0.6	0.12	0.6	0.13	1.4	0.33	1.3	0.45	2.7	0.43	

Hispanic	3.0	0.33	3.0	0.43	3.3	0.57	2.8	0.76	3.8	0.63	

**Veteran Status**											

Yes	13.2	0.47	13.5	0.68	12.9	1.00	13.0	1.13	12.0	0.89	0.749

No	86.8	0.47	86.5	0.68	87.1	1.00	87.0	1.13	88.0	0.89	

**Marital Status**											

Married	64.4	0.74	63.9	1.07	61.6	1.60	59.5	1.85	53.1	1.43	<.001

Divorced/Separated	8.6	0.35	11.4	0.62	13.2	0.90	14.9	1.18	19.7	1.13	

Widowed	12.6	0.37	8.5	0.44	7.2	0.56	8.0	0.76	5.3	0.46	

Never Married	12.7	0.69	14.0	0.94	15.5	1.60	15.1	1.65	16.7	1.26	

Unmarried Couple	1.7	0.23	2.2	0.41	2.6	0.42	2.6	0.53	5.2	0.63	

**Education**											

Never Attended School	0.3	0.09	0.5	0.22	0.2	0.10	0.0	0.00	0.2	0.08	<.001

<High School	14.1	0.68	13.8	0.93	15.2	1.45	12.0	1.39	20.2	1.38	

High School Graduate	36.0	0.71	35.6	1.03	35.7	1.50	35.1	1.83	32.2	1.31	

Some College	29.5	0.68	30.2	1.00	31.4	1.50	34.2	1.77	33.7	1.30	

College	20.1	0.51	20.0	0.73	17.5	0.90	18.8	1.19	13.8	0.77	

**Income**											

<$15,000	8.5	0.44	8.7	0.6	9.0	0.83	11.6	1.32	15.7	0.97	<.001

$15,000 to less than $25,000	20.3	0.69	19.9	0.98	20.8	1.38	19.0	1.59	25.1	1.42	

$25,000 to less than $35,000	14.7	0.56	15.4	0.86	15.3	1.20	14.7	1.41	12.2	0.95	

$35,000 to less than $50,000	18.5	0.63	16.8	0.81	16.5	1.03	17.4	1.53	16.5	1.03	

>$50,000	38.0	0.78	39.2	1.12	38.4	1.55	37.4	1.88	30.5	1.35	

**Employment Status**											

Employed or Self-Employed	50.5	0.75	54.1	1.08	56.1	1.57	56.4	1.83	54.4	1.40	<.001

Out of Work	3.2	0.30	6.0	0.59	7.1	0.95	8.4	1.10	10.9	0.93	

Homemaker/Student	8.8	0.47	8.3	0.68	9.3	1.18	8.6	1.16	8.7	0.78	

Retired	32.0	0.63	25.2	0.82	19.2	0.98	17.4	1.16	10.2	0.64	

Unable to Work	5.4	0.36	6.5	0.51	8.3	0.80	9.2	1.00	15.9	1.01	

**URBAN** **(n=36,141)**	**ACE Score 0** **n=15,457** **(%)**	**SE**	**ACE Score 1** **n=8,057** **(%)**	**SE**	**ACE Score 2** **n=4,510** **(%)**	**SE**	**ACE Score 3** **n=2,823** **(%)**	**SE**	**ACE Score 4+** **n=5,294** **(%)**	**SE**	**p-value**

**Age (years)**											

18-24	6.2	0.55	8.3	0.79	1.2	0.81	6.3	1.07	9.3	0.97	<.001

25-34	8.4	0.43	9.1	0.58	1.0	0.74	10.9	1.12	15.1	0.86	

35-44	14.1	0.50	17.8	0.77	1.0	0.73	19.9	1.30	20.7	1.00	

45-54	19.1	0.54	21.9	0.82	1.0	0.76	23.7	1.32	25.9	0.98	

55-64	20.3	0.51	20.1	0.66	0.8	0.70	23.1	1.22	18.9	0.79	

≥65	31.8	0.57	22.9	0.7	0.9	0.60	16.1	0.95	9.9	0.55	

**Sex**											

Male	46.5	0.69	47.5	0.96	47.4	1.30	41.6	0.39	37.5	1.18	<.001

Female	53.5	0.69	52.5	0.96	52.6	1.30	58.4	0.45	62.5	1.18	

**Race/Ethnicity**											

White, non-Hispanic (NH)	83.4	0.54	81.2	0.85	79.6	1.18	1.2	0.52	78.3	1.07	<.001

Black, NH	7.2	0.35	9.0	0.58	8.3	0.70	1.0	0.40	9.8	0.79	

Asian, NH	4.4	0.34	3.4	0.45	1.9	0.33	0.3	0.08	1.1	0.28	

Native Hawaiian/Other Pacific Islander, NH	0.1	0.03	0.1	0.06	0.6	0.36	0.1	0.19	0.4	0.20	

American Indian/Alaska Native, NH	0.6	0.10	0.8	0.14	1.4	0.33	0.3	0.60	2.7	0.54	

Other, NH	0.6	0.12	0.4	0.09	0.9	0.22	0.2	0.68	0.6	0.12	

Multi, NH	0.6	0.09	0.8	0.13	1.8	0.39	0.2	0.34	3.0	0.42	

Hispanic	3.0	0.27	4.3	0.55	5.6	0.86	0.6	0.13	4.2	0.48	

**Veteran Status**											

Yes	13.8	0.44	11.8	0.50	12.7	0.75	13.3	1.01	11.7	0.71	0.024

No	86.2	0.44	88.2	0.50	87.4	0.75	86.7	1.01	88.3	0.71	

**Marital Status**											

Married	65.3	0.66	61.9	0.95	58.6	1.32	60.1	1.55	54.5	1.17	<.001

Divorced/Separated	8.9	0.31	10.2	0.47	10.9	0.63	14.2	1.11	16.0	0.68	

Widowed	10.8	0.31	7.9	0.37	7.1	0.50	6.9	0.70	4.2	0.32	

Never Married	13.2	0.60	17.6	0.91	19.7	1.30	14.8	1.19	20.0	1.12	

Unmarried Couple	1.8	0.20	2.4	0.33	3.7	0.58	4.0	0.64	5.4	0.61	

**Education**											

Never Attended School	0.2	0.06	0.1	0.07	0.0	0.02	0.5	0.29	0.0	0.02	<.001

<High School	8.5	0.46	9.5	0.69	9.2	0.88	11.2	1.19	13.7	1.01	

High School Graduate	28.1	0.62	27.5	0.86	26.9	1.24	28.0	1.51	27.1	1.02	

Some College	29.8	0.64	32.0	0.92	34.1	1.22	32.8	1.45	37.0	1.13	

College	33.4	0.60	30.8	0.8	29.8	1.06	27.5	1.22	22.1	0.84	

**Income**											

<$15,000	5.2	0.29	6.8	0.54	7.1	0.69	8.2	0.79	11.6	0.66	<.001

$15,000 to less than $25,000	14.3	0.51	13.1	0.76	14.1	1.05	16.4	1.32	19.8	1.07	

$25,000 to less than $35,000	12.3	0.47	11.4	0.62	12.8	0.94	11.1	1.01	11.7	0.86	

$35,000 to less than $50,000	15.8	0.54	14.5	0.65	14.3	0.93	15.7	1.23	14.0	0.79	

>$50,000	52.4	0.74	54.3	1.02	51.7	1.39	48.6	1.62	42.9	1.19	

**Employment Status**											

Employed or Self-Employed	52.0	0.68	55.8	0.95	57.5	1.26	56.7	1.56	53.9	1.16	<.001

Out of Work	4.0	0.26	6.0	0.48	6.7	0.63	8.8	0.96	11.2	0.72	

Homemaker/Student	10.2	0.51	10.9	0.73	11.0	0.96	9.0	1.01	12.4	0.87	

Retired	30.1	0.56	22.5	0.70	18.9	0.85	16.9	0.99	10.6	0.58	

Unable to Work	3.8	0.23	4.8	0.39	6.0	0.52	8.6	0.97	11.9	0.72	

**Table 3:** Logistic regression showing impact of ace scores on health outcomes among adults aged 18+ in rural and urban areas of the United States, 2011-2012 BRFSS, 9 states.

**Rural Areas (n=29,521)**					

	**Score 0** **n=13521**	**Score 1** **n=6320**	**Score 2** **n=3446**	**Score 3** **n=2160**	**Score ≥4** **n=4074**

**Activity Limitation Due to Physical, Mental, or Emotional (ref=no)**					

Unadjusted Prevalence (SE)	20.19 (0.55)	23.55 (0.86)	26.38 (1.27)	30.20 (1.68)	38.01 (1.34)

Unadjusted Odds Ratio (OR)	1.00 (Reference)	1.22 (1.08, 1.37)^**∗**^	1.42 (1.23, 1.64)^**∗**^	1.71 (1.44, 2.03)^**∗**^	2.42 (2.13, 2.76)^**∗**^

Adjusted Odds Ratio (AOR)^1^	1.00 (Reference)	1.36 (1.18, 1.56)^**∗**^	1.59 (1.34, 1.88)^**∗**^	1.90 (1.54, 2.35)^**∗**^	2.66 (2.26, 3.13)^**∗**^

**General Health Rated as Fair/Poor (ref=excellent/very good/good)**					

Unadjusted Prevalence (SE)	16.56 (0.53)	17.46 (0.78)	19.11 (1.11)	20.95 (1.46)	27.92 (1.26)

Unadjusted Odds Ratio (OR)	1.00 (Reference)	**1.07 (0.94, 1.22)**	1.20 (1.02, 1.41)^**∗∗**^	1.35 (1.11, 1.62)^**∗**^	1.97 (1.70, 2.27)^**∗**^

Adjusted Odds Ratio (AOR)^1^	1.00 (Reference)	1.19 (1.02, 1,39)^**∗∗**^	1.36 (1.12, 1.66)^**∗**^	1.46 (1.15, 1.85)^**∗**^	2.02 (1.67, 2.44)^**∗**^

**Ever Had Diabetes (ref=no diabetes)**					

Unadjusted Prevalence (SE)	11.68 (0.44)	11.69 (0.66)	11.74 (0.88)	12.58 (1.15)	10.82 (0.81)

Unadjusted Odds Ratio (OR)	1.00 (Reference)	1.00 (0.86, 1.16)	1.01 (0.83, 1.21)	1.09 (0.87, 1.36)	0.92 (0.76, 1.10)

Adjusted Odds Ratio (AOR)^1^	1.00 (Reference)	1.17 (0.99, 1.38)	1.22 (0.99, 1.51)	1.36 (1.06, 1.73)^**∗∗**^	1.12 (0.90, 1.41)

**Ever Had Stroke (ref=no stroke)**					

Unadjusted Prevalence (SE)	3.74 (0.25)	3.77 (0.40)	2.82 (0.39)	3.69 (0.57)	3.81 (0.46)

Unadjusted Odds Ratio (OR)	1.00 (Reference)	1.01 (0.78, 1.30)	0.75 (0.55, 1.02)	0.99 (0.70, 1.39)	1.02 (0.70, 1.35)

Adjusted Odds Ratio (AOR)^1^	1.00 (Reference)	1.26 (0.94, 1.68)	0.90 (0.62, 1.30)	1.22 (0.81, 1.85)	1.27 (0.90, 1.79)

**Ever Diagnosed with Heart Attack (ref=no heart attack)**					

Unadjusted Prevalence (SE)	6.08 (0.32)	5.95 (0.45)	5.71 (0.64)	5.81 (0.70)	6.61 (0.75)

Unadjusted Odds Ratio (OR)	1.00 (Reference)	0.98 (0.81, 1.19)	0.94 (0.72, 1.21)	0.95 (0.72, 1.25)	1.09 (0.84, 1.42)

Adjusted Odds Ratio (AOR)^1^	1.00 (Reference)	1.20 (0.96, 1.49)	1.35 (1.01, 1.82)^**∗∗**^	1.31 (0.96, 1.78)	1.94 (1.44, 2.62)^**∗**^

**Ever Had Angina or Coronary Heart Disease (ref=no heart disease)**					

Unadjusted Prevalence (SE)	5.40 (0.29)	6.67 (0.51)	6.7 (0.76)	5.6 (0.65)	6.8 (0.74)

Unadjusted Odds Ratio (OR)	1.00 (Reference)	1.25 (1.03, 1.52)^**∗∗**^	1.27 (0.98, 1.65)	1.03 (0.79, 1.35)	1.27 (0.98, 1.64)

Adjusted Odds Ratio (AOR)^1^	1.00 (Reference)	1.65 (1.33, 2.04)^**∗**^	1.99 (1.47, 2.69)^**∗**^	1.52 (1.12, 2.06)^**∗**^	2.44 (1.84, 3.21)^**∗**^

**Ever Had Asthma (ref=no asthma)**					

Unadjusted Prevalence (SE)	8.38 (0.47)	10.64 (0.70)	11.07 (0.89)	13.87 (0.13)	18.29 (0.11)

Unadjusted Odds Ratio (OR)	1.00 (Reference)	1.30 (1.08, 1.57)^**∗**^	1.36 (1.10, 1.69)^**∗**^	1.76 (1.39, 2.23)^**∗**^	2.45 (2.03, 2.95)^**∗**^

Adjusted Odds Ratio (AOR)^1^	1.00 (Reference)	1.22 (1.00, 1.48)	1.22 (0.96, 1.56)	1.71 (1.32, 2.21)^**∗**^	1.84 (1.48, 2.29)^**∗**^

**Poor Mental Health (ref=good mental health)**					

Unadjusted Prevalence (SE)	27.20 (1.42)	30.3 (1.89)	29.51 (2.33)	37.24 (2.97)	46.03 (1.98)

Unadjusted Odds Ratio (OR)	1.00 (Reference)	1.17 (0.93, 1.46)	1.12 (0.86, 1.45)	1.59 (1.19, 2.11)^**∗**^	2.28 (1.85, 2.82)^**∗**^

Adjusted Odds Ratio (AOR)^1^	1.00 (Reference)	1.20 (0.94, 1.53)	1.19 (0.90, 1.57)	1.62 (1.17, 2.24)^**∗**^	1.90 (1.48, 2.45)^**∗**^

**Urban Areas (n=36,141)**					

	**Score 0** **n=15457**	**Score 1** **n=8057**	**Score 2** **n=4510**	**Score 3** **n=2823**	**Score≥4** **n=5294**

**Activity Limitation Due to Physical, Mental, or Emotional (ref=no)**					

Unadjusted Prevalence (SE)	18.67 (0.46)	23.08 (0.77)	25.65 (1.02)	27.69 (1.31)	35.73 (1.11)

Unadjusted Odds Ratio (OR)	1.00 (Reference)	1.31 (1.18, 1.45)^**∗**^	1.50 (1.33, 1.70)^**∗**^	1.67 (1.45, 1.92)^**∗**^	2.42 (2.17, 2.71)^**∗**^

Adjusted Odds Ratio (AOR)^1^	1.00 (Reference)	1.51 (1.33, 1.71)^**∗**^	1.75 (1.52, 2.02)^**∗**^	1.90 (1.62, 2.23)^**∗**^	2.62 (2.29, 3.00)^**∗**^

**General Health Rated as Fair/Poor (ref=excellent/very good/good)**					

Unadjusted Prevalence (SE)	13.43 (0.47)	14.00 (0.66)	15.25 (0.87)	20.48 (1.25)	23.06 (0.94)

Unadjusted Odds Ratio (OR)	1.00 (Reference)	1.05 (0.92, 1.19)	1.16 (1.00, 1.35)	1.66 (1.40, 1.96)^**∗**^	1.93 (1.70, 2.19)^**∗**^

Adjusted Odds Ratio (AOR)^1^	1.00 (Reference)	1.07 (0.92, 1.25)	1.21 (1.00, 1.46)	1.77 (1.45, 2.15)^**∗**^	1.78 (1.50, 2.11)^**∗**^

**Ever Had Diabetes (ref=no diabetes)**					

Unadjusted Prevalence (SE)	10.76 (0.37)	10.41 (0.54)	11.37 (0.75)	13.64 (1.10)	11.55 (0.66)

Unadjusted Odds Ratio (OR)	1.00 (Reference)	0.96 (0.84, 1.10)	1.06 (0.90, 1.25)	1.31 (1.07, 1.60)^**∗**^	1.08 (0.94, 1.25)

Adjusted Odds Ratio (AOR)^1^	1.00 (Reference)	1.09 (0.94, 1.27)	1.36 (1.14, 1.63)^**∗**^	1.47 (1.18, 1.84)^**∗**^	1.39 (1.16, 1.65)^**∗**^

**Ever Had Stroke (ref=no stroke)**					

Unadjusted Prevalence (SE)	2.89 (0.18)	3.09 (0.28)	2.84 (0.39)	4.00 (0.73)	3.37 (0.34)

Unadjusted Odds Ratio (OR)	1.00 (Reference)	1.07 (0.86, 1.34)	0.98 (0.73, 1.33)	1.40 (0.95, 2.07)	1.17 (0.92, 1.49)

Adjusted Odds Ratio (AOR)^1^	1.00 (Reference)	1.24 (0.97, 1.59)	1.22 (0.88, 1.69)	1.47 (0.97, 2.23)	1.48 (1.11, 1.97)^**∗**^

**Ever Diagnosed with Heart Attack (ref=no heart attack)**					

Unadjusted Prevalence (SE)	5.15 (0.27)	5.11 (0.44)	4.17 (0.45)	5.71 (0.80)	4.72 (0.41)

Unadjusted Odds Ratio (OR)	1.00 (Reference)	0.99 (0.80, 1.22)	0.80 (0.63, 1.02)	1.11 (0.82, 1.52)	0.91 (0.74, 1.12)

Adjusted Odds Ratio (AOR)^1^	1.00 (Reference)	1.18 (0.94, 1.49)	1.05 (0.80, 1.37)	1.43 (1.03, 2.00)^**∗∗**^	1.39 (1.08, 1.79)^**∗∗**^

**Ever Had Angina or Coronary Heart Disease (ref=no heart disease)**					

Unadjusted Prevalence (SE)	5.22 (0.27)	5.14 (0.41)	4.84 (0.48)	5.06 (0.77)	4.58 (0.39)

Unadjusted Odds Ratio (OR)	1.00 (Reference)	0.98 (0.81, 1.20)	0.92 (0.74, 1.16)	0.97 (0.69, 1.35)	0.87 (0.71, 1.07)

Adjusted Odds Ratio (AOR)^1^	1.00 (Reference)	1.18 (0.94, 1.47)	1.23 (0.95, 1.59)	1.32 (0.93, 1.88)	1.41 (1.10, 1.81)^**∗**^

**Ever Had Asthma (ref=no asthma)**					

Unadjusted Prevalence (SE)	8.30 (0.40)	11.59 (0.64)	13.07 (0.90)	13.61 (1.11)	17.62 (0.88)

Unadjusted Odds Ratio (OR)	1.00 (Reference)	0.98 (0.81, 1.20)	0.92 (0.74, 1.16)	0.97 (0.69, 1.35)	0.87 (0.71, 1.07)

Adjusted Odds Ratio (AOR)^1^	1.00 (Reference)	1.40 (1.17, 1.66)^**∗**^	1.51 (1.24, 1.84)^**∗∗**^	1.55 (1.25, 1.91)^**∗**^	1.76 (1.48, 2.10)^**∗**^

**Poor Mental Health (ref=good mental health)**					

Unadjusted Prevalence (SE)	25.13 (1.26)	25.63 (1.61)	28.84 (2.01)	31.50 (2.15)	42.90 (1.62)

Unadjusted Odds Ratio (OR)	1.00 (Reference)	1.03 (0.83, 1.27)	1.21 (0.96, 1.52)	1.37 (1.08, 1.73)^**∗**^	2.24 (1.86, 2.69)^**∗**^

Adjusted Odds Ratio (AOR)^1^	1.00 (Reference)	1.07 (0.85, 1.35)	1.12 (0.85, 1.47)	1.30 (1.00, 1.70)	1.91 (1.55, 2.34)^**∗**^

^1^Adjusted  for age, sex, race/ethnicity, veteran status, marital status, employment status, education, and family income. ^*∗*^p<.01, ^*∗∗*^p<.05.
